# The effects of procrastination on physical activity among Chinese university students: the chain-mediated effects of time management disposition and exercise motivation

**DOI:** 10.3389/fpsyg.2024.1433880

**Published:** 2024-11-15

**Authors:** Yuan Zhang, Maoshen Tian, Jian Yang, Yue Xi, Zhihui Li, Lin Wang

**Affiliations:** ^1^College of Physical Education and Health, East China Normal University, Shanghai, China; ^2^College of Physical Education and Health, Changsha Medical University, Changsha, China; ^3^College of Humanity and Law, Henan Agricultural University, Zhengzhou, China

**Keywords:** procrastination, physical activity, time management disposition, exercise motivation, college students

## Abstract

**Objectives:**

Grounded in self-determination theory (SDT), the procrastination-health model, and the mechanism model of exercise persistence, this study examined the effects of procrastination on physical activity and the mechanism of its action in Chinese college students.

**Design:**

This study employed a cross-sectional design.

**Methods:**

A total of 957 Chinese university students (Mage = 20.26, SD = 1.07) completed questionnaires. The Aitken Procrastination Questionnaire, the Adolescent Time Management Dispositions Scale (ATMDS), the Modified Physical Activity Motivation Measure-Revised (MPAM-R), and the Physical Activity Rating Scale (PARS-3) were used to measure procrastination, time management disposition, exercise motivation, and physical activity.

**Results:**

Procrastination, time management disposition, exercise motivation, and physical activity were each significantly correlated, and procrastination was a significant negative predictor of physical activity. The mediating effect of time management disposition and exercise motivation in the effect of procrastination on physical activity was significant, and the mediating effect accounted for 44.65% of the total effect. Three paths were specifically included: first, the separate mediating effect of time management disposition, second, the separate mediating effect of exercise motivation, and third, the chain mediating effect of time management disposition and exercise motivation.

**Conclusion:**

This study reveals the mechanism of action by which procrastination influences physical activities through time management disposition and exercise motivation in Chinese college students. The findings provide guiding recommendations for further promoting greater participation in physical activities among college students.

## 1 Introduction

Since the 21st century, physical inactivity has become the fourth most important risk factor for death globally after hypertension, smoking, and hyperglycemia (Bull et al., 2020). A recent study found that nearly one-third (31%) of the world’s adult population, or approximately 1.8 billion adults, are physically inactive (Strain et al., 2024). A large number of studies have demonstrated the effects of active physical activity on physical and mental health, such as preventing cognitive decline, improving musculoskeletal function, and alleviating anxiety, depression, and other negative emotions (Guo et al., 2020; Zhang et al., 2020; Liu, 2020; Puts et al., 2017; Josefsson et al., 2014; Singh et al., 2023), and lowering the incidence of chronic non-communicable diseases such as type 2 diabetes and cardiovascular disease (John-Henderson et al., 2021). The college student population is a group at the forefront of new technologies and ideas in society, representing youth and vitality, and is a major driver of social progress. However, a large number of studies have shown that during the transition from adolescence to early adulthood, there is a sharp decline in physical activity (Kwan et al., 2012; Telama and Yang, 2000), followed by sedentary behavior (Bull et al., 2020), obesity (Jebeile et al., 2022), etc., which seriously affects the physical and mental health of college students. Currently, various countries have begun to actively adopt various ways to promote physical activity participation. In China, policy documents such as the “14th Five-Year Plan for Sports Development,” “Physical Activity Guidelines for the Chinese Population (2021),” and “Opinions on Deepening the Integration of Physical Education and Sports to Promote the Healthy Development of Adolescents” have been issued one after the other, which have provided a scientific basis for the physical activity of different groups of people. Many Chinese scholars have made suggestions on the promotion of comprehensive health development of adolescents from personal, interpersonal, environmental, and policy perspectives. There are also many Chinese scholars who have conducted research on promoting the overall healthy development of adolescents and avoiding the potential health risks brought about by insufficient physical activity from personal, interpersonal, environmental, and policy perspectives (Wu and Yang, 2023). Therefore, as physical activity is an important way to maintain the physical and mental health of college students, it is of great practical significance to explore the factors affecting college students’ physical activity and promote college students’ active participation in physical activity on this basis.

Previous research has found that procrastination is a very common personality trait and behavioral tendency in modern society (Kim and Seo, 2015). Some scholars believe that procrastination is a behavioral tendency related to the degree of aversion to what is to be done and the fear of failure (Wolters, 2003), which belongs to the failure of self-regulation (Steel, 2007), and is a kind of avoidance behavior toward tasks. Some scholars believe that procrastination is a personality trait characterized by a consistent and persistent delay in starting or completing a task (Ferrari and Tice, 2000). Procrastination is defined as a novel and plausible perspective to explain failures of self-regulation in the context of health behaviors (Kroese and de Ridder, 2016). Procrastination has been found to be a representative problematic behavior in college students’ learning that can directly or indirectly affect college students’ academic performance through factors such as academic anxiety and stress (Kim and Seo, 2015; Manchado Porras and Hervias Ortega, 2021; Zhao et al., 2022). In addition, procrastination produces problems related to problematic cell phone use, Internet addiction, and more (Gong et al., 2021). The procrastination-health model states that procrastination affects health not only through stress but also indirectly through delayed health behaviors and disease prevention (Sirois et al., 2003). For example, bedtime procrastination is a sign of a lack of self-regulation, which can lead to sleep deprivation and affect the quality of sleep (Exelmans and Van den Bulck, 2021; Kroese et al., 2014). Physical activity is a healthy behavior in which people can be physically active enough to enhance their fitness and have a positive emotional experience, but engaging in strenuous physical activity may produce an unpleasant experience, which then may lead to procrastination (Ren et al., 2023; Shi et al., 2021). Research indicates that college students with a high tendency to procrastinate often struggle with self-management of exercise. They tend to have less clarity in their intentions and planning for physical activity, leading to increased randomness and inertia in their exercise behaviors, which negatively impacts their overall exercise performance. Therefore, reducing procrastination behavior among college students can help to promote active physical activity among college students. Overall, procrastination, a common personality trait and behavioral tendency among college students, has a significant impact on their health behaviors (e.g., physical activity), but it is unclear how procrastination can influence intrinsic psychological factors that can promote physical activity among college students. Previous studies have found a strong relationship between time management disposition and exercise motivation and physical activity; therefore, the present study intends to examine whether procrastination can have an effect on physical activity through the mediation of time management disposition and exercise motivation. Based on this, this study proposes the first hypothesis:

*H1*: Procrastination has a negative and significant effect on physical activity.

### 1.1 The mediating role of time management dispositions

Time management tendency is the psychological and behavioral characteristics of individuals in terms of the function and value of time, and the way they use time, which includes three dimensions: the sense of time value, the sense of time control, and the sense of time efficacy (Huang and Zhang, 2001b). Higher time management disposition indicates that individuals are more certain of the value of time, have higher awareness and ability to monitor time in their study and life, and have more confidence in their own time management. It has been shown that time management is positively related to perceived time control, job satisfaction, and psychological and social adjustment and negatively related to stress (Claessens et al., 2007; Häfner et al., 2014) and directly affects job performance, academic achievement, and procrastination behavior (Wolters and Brady, 2021). Numerous studies have shown that time management is directly linked to variables closely related to the quality of life, such as healthy eating and physical activity (Indreica et al., 2022), and is a very worthwhile personal factor to consider (Connelly et al., 2015; Das and Evans, 2014; Heard et al., 2017). Strong time management skills are essential for ensuring regular participation in physical activity (Zhu, 2018). Conversely, a lack of time and irrational allocation and utilization of time are significant factors that can affect weight loss (Delahanty et al., 2019) and adversely affect overall performance in physical activities (Rebar et al., 2019). Self-regulation theory suggests that performing healthful activities requires a certain level of time regulation and self-control and that the tendency to procrastinate on time commitment for physical activity is related to the ability to self-manage time (Codina et al., 2020). College students are in the sensitive period of social adaptation and development, but also a critical period of personality and executive function improvement, procrastination, once formed, will become a relatively stable personality trait of the individual. Procrastination individuals tend to have lower self-control ability is lower, difficult to better manage and allocate time, is very likely to affect the participation of physical activities. Therefore, a second hypothesis is proposed:

*H2*: Time management disposition has a significant mediating effect between procrastination and physical activity, that is, procrastination reduces physical activity by reducing the level of time management disposition.

### 1.2 The mediating role of exercise motivation

Exercise motivation is the internal psychological motivation that inspires and sustains individuals to engage in physical activity. Self-determination theory (SDT) is a motivational process theory about individual self-determination behavior, which is widely used in the fields of education (Behzadnia et al., 2023), management (Huang et al., 2023), sports (Liu, 2020), etc. This theory suggests that an individual’s motivation is on a continuum from intrinsic motivation to extrinsic motivation and non-motivation (Behzadnia et al., 2018) and that intrinsic motivation positively influences adherence and persistence of behavior (Deci and Ryan, 1985). Exercise motivation is the direct impetus that drives individuals to engage in physical activity; it explains that individuals can satisfy certain needs by engaging in physical activity and reflects the reasons why individuals want to participate in physical activity. According to the source of motivation (internal/external) and its effect on exercise behavior (strong/weak), it can be classified as ability motivation, health motivation, fun motivation, social motivation, and appearance motivation (Chen et al., 2013). There are a number of studies that have examined exercise behavior from the perspective of self-determination theory, and these studies have validated the role of exercise motivation as a predictor of exercise behavior (Thogersen-Ntoumani and Ntoumanis, 2006). At the same time, the satisfaction of basic needs can stimulate more self-determined motivation. Exercise motivation can directly affect sedentary behavior (Esmaeilzadeh et al., 2022), social adaptation (Chen et al., 2022), etc., and different levels of exercise motivation are also capable of positively influencing the short-term emotional benefits of physical activity (Zhang et al., 2019). The effect of procrastination on behavioral execution and persistence cannot be ignored but also indirectly affects behavior through the mediating role of psychological variables that are directional to behavior. Individuals with high procrastination tend to respond to events with higher levels of discretion and inertia, which can reduce the degree of self-determination in physical exercise motivation (Wang et al., 2015). In addition, procrastination is manifested as a deviation between behavioral intention and actual behavior. Behavioral intention is a motivational factor for an individual to engage in a particular behavior, reflecting the level of effort an individual is willing to put in, and is a direct factor in behavioral decisions. Therefore, procrastination may affect the occurrence of physical activity behaviors by reducing physical activity motivation. Based on the above, this study proposes the third hypothesis:

*H3*: Exercise motivation has a significant mediating effect between procrastination and physical activity, that is, procrastination reduces physical activity by reducing the level of exercise motivation.

### 1.3 Chain mediation of time management disposition and exercise motivation

Both time management disposition and exercise motivation can serve as internal psychological traits of individuals that promote the onset and persistence of exercise behavior (Li et al., 2021). The mechanism model of exercise persistence emphasizes the important role of exercise motivation in the generation and persistence of exercise behavior, pointing out that physical activity is realized through the path of “social environment → individual →cognitive decision-making → commitment →behavior,” in which exercise motivation is the direct antecedent of the psychological mechanism of exercise commitment, which can be determined by the formation of behavioral intentions and produce actual physical activity (Chen et al., 2006). In addition, exercise motivation also acts as an important mediator in personality traits and emotional benefits of exercise (Jiang et al., 2015), and perceived social support and exercise behavior (Hui et al., 2022). Among them, personality traits are considered to be important determinants of exercise motivation. Previous research has shown that there is also a strong link between these two internal resources, with time management positively predicting achievement motivation (Song and Chen, 2016; Yang et al., 2014). Time management disposition involves an individual’s awareness of the meaning of time, from which the individual develops a behavioral tendency in time management. In the field of sports, individuals with high levels of time management disposition have strong self-control and a correct perception of the positive effects of self-imposed physical activity on their health and thus are likely to show strong exercise motivation. However, few studies have considered the combined effects of time management disposition and exercise motivation when focusing on the influencing factors of physical activity. Therefore, based on hypotheses H1, H2, and H3, this study proposes a fourth hypothesis:

*H4*: Time management disposition and exercise motivation have a chain-mediated effect between procrastination and physical activity, that is, procrastination reduces physical activity by sequentially reducing time management disposition and exercise motivation.

**Figure 1:**
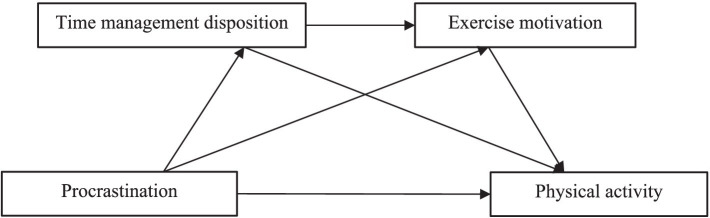
Hypothetical model of the relationship between procrastination, time management tendencies, exercise motivation, and physical activity in college students.

Based on this, this study aims to explore the influence of procrastination on college students’ physical activity and also analyze the chain-mediated role of time management disposition and exercise motivation between procrastination and physical activity, so as to provide a reference for exploring the influence mechanism of college students’ physical activity to promote college students’ active participation in physical activity. The chain mediation model is shown in Figure 1.

## 2 Materials and methods

### 2.1 Participants and procedures

The study was conducted on Chinese college students in six schools in the cities of Beijing, Shanghai, and Zhengzhou, following the principle of convenience sampling. All subjects were able to participate in school studies and sports activities normally and had no significant physical or mental diseases. A total of 1,022 questionnaires were recovered from the survey, and 957 questionnaires met the requirements after elimination to ensure the accuracy of the data, with an effective rate of 93.64%. The subjects who participated in the survey were divided by gender, 411 male (42.947%) and 546 female (57.05%) students; and by grade, 299 freshmen (31.24%), 264 sophomores (27.59%), 245 juniors (25.60%), and 149 seniors (15.57%). The mean age of the participants was 20.26 years (SD = 1.07; min = 18, max = 23). The questionnaires were rejected on the basis of the following: (1) the answer time was less than 240 s and more than 900 s; (2) the answer to the reverse question was contradictory; and (3) the answers were all consistent or had a certain pattern (e.g., “11111…” or “123123…”).

The questionnaires were distributed and collected through the Questionnaire Star platform, a professional online survey tool known for its speed, ease of use, and low cost, making it widely used in surveys and research in China. The study was approved by the Ethics Committee of East China Normal University in China (HR515-2019), and according to the requirements of the ethical review system, this study did not require participants to sign an informed consent form for ethical review, even so, information about the content of the questionnaire and a reminder stating that the survey was anonymous and that the results would be used only for scientific research and would not pose any risk to their daily lives. At the same time, participation in the survey was completely voluntary, and participants could abandon or terminate the questionnaire at any time during the administration process according to their own wishes.

### 2.2 Methodology

#### 2.2.1 Aitken procrastination questionnaire

The Aitken Procrastination Questionnaire, revised by Chinese scholars Xiaoli Chen, Xiaoyang Dai, and Qin Dong, was used to assess the procrastination behavior of college students. Students responded to the 19-item of the Aitken procrastination questionnaire (e.g., “I always wait until the last minute to do things”). Students responded to a scale ranging from 1 (*not conforming at all*) to 5 (*conforming completely*). In this study, Cronbach’s *α* was 0.847, and the validated factor analysis fit indices were as follows: χ^2^/df = 5.929, GFI = 0.908, AGFI = 0.833, TLI = 0.865, NFI = 0.862, CFI = 0.882, RMSEA = 0.072, SRMR = 0.0734, KMO = 0.897, and Bartlett’s test showed *p* < 0.001. These results indicate good reliability and validity of the scale.

#### 2.2.2 Adolescence time management disposition scale (ATMDS)

The Adolescence Time Management Disposition Scale (ATMDS) developed by Chinese scholar (Huang and Zhang, 2001a) was used as a tool to evaluate adolescents’ personality traits in terms of time mastery and control. This scale assesses time management disposition in three dimensions, namely, the sense of time value (10 items, e.g., “I think the saying ‘An ounce of time is worth an ounce of gold’ is true”), the sense of time control (24 items, e.g., “I always spend a lot of time doing important work”), and the sense of time efficacy (10 items, e.g., “I always test my plans against the accomplishment of my goals”), with a total of 44 items. The items were rated using a 7-point scale (1 = *not conforming at all* and 7 = *conforming completely*). The higher the score, the stronger the time management disposition, and the better the ability to rationally manage and utilize time. In this study, Cronbach’s alpha was 0.939, and the fit index of validated factor analysis fit indices were as follows: χ^2^/df = 5.047, GFI = 0.807, AGFI = 0.787, TLI = 0.813, NFI = 0.789, CFI = 0.823, RMSEA = 0.065, SRMR = 0.0824, KMO = 0.956, and Bartlett’s test showed *p* < 0.001. These results indicate good reliability and validity of the scale.

#### 2.2.3 The simplified version of the motives for physical activities measure-revised scale (MPAM-R)

Students’ exercise motivation was assessed through the Simplified Version of the Motives for Physical Activities Measure-Revised Scale (MPAM-R), which was revised by Chinese scholar Chen et al. (2013). The MPAM-R assesses college students’ exercise motivation in five dimensions, namely, health motivation (three items, e.g., “I want to be fit”), appearance motivation (three items, e.g., “I want to lose weight”), fun motivation (three items, e.g., “I want to participate in entertaining activities”), ability motivation (three items, e.g., “I want to gain new motor skills”), and social motivation (three items, e.g., “I want to meet some new friends”). The items were rated using a 5-point scale (1 = *not conforming at all*, 5 = *conforming completely*). In this study, Cronbach’s *α* was 0.952, and the validated factor analysis fit indices were as follows: χ^2^/df = 8.651, GFI = 0.893, AGFI = 0.856, TLI = 0.933, NFI = 0.937, CFI = 0.943, RMSEA = 0.089, SRMR = 0.040, KMO = 0.964, and Bartlett’s test *p* < 0.001. These results indicate good reliability and validity of the scale.

#### 2.2.4 Physical activity rating scale (PARS-3)

Students’ physical activity was assessed through the Physical Activity Rating Scale (PARS-3), which was revised by Chinese scholar Liang (1994). Participants were asked to rate the frequency (e.g., “How many times a month do you do the above physical activities”), duration (e.g., “How many minutes at a time do you perform physical activity at the above intensities”), and intensity (e.g., “How intensely do you exercise”) of their bodily movements from 1 to 5. The total score for physical activities was the product of the scores on frequency, duration (minus 1), and intensity. The scale has good psychometric properties and has been widely used for young adults in Chinese culture (Feng et al., 2015; Ge and He, 2022). In this study, the amount of exercise was used as an assessment index of participants’ physical activity. Cronbach’s α in this study was 0.952.

### 2.3 Data analyses

In this study, SPSS 26.0 and Amos 23.0 were used to process and analyze the recovered questionnaire data. First, internal consistency analysis and validation factor analysis were used to test the reliability and validity of the scales. Second, descriptive statistics and correlation analysis were used to examine the relationship between procrastination, time management disposition, exercise motivation, and physical activity. Third, the SPSS Process 3.3 plug-in was used, and the Bootstrap method was chosen to analyze the indirect effect of procrastination affecting the amount of physical activity (chain-mediated effect of time management disposition and exercise motivation). Setting the independent variable (X) = procrastination, mediating variable (M1) = time management disposition, mediating variable (M2) = exercise motivation, control variables = gender, and dependent variable (Y) = physical activity, selecting Model 6 based on templates, and repeating the sample of 5,000, with the default 95% confidence interval, to conduct the analysis of the chained mediation model effect. In addition, AMOS 26.0 was used in this study to validate the reasonableness of the model, and the results showed good model fit indicators for the hypothesized model: χ^2^/df = 2.145, CFI = 0.996, TLI = 0.993, RMSEA = 0.035, and SRMR = 0.0169.

## 3 Results

### 3.1 Common methodology bias control and testing

Since all the data in this study came from the self-reports of Chinese university students, the results may be affected by common methodological biases. Therefore, measures such as separating different questionnaires, reverse scoring some questions, and emphasizing the confidentiality of data were taken for prior procedural control during the study design and data collection process. In addition, the study used the Harman one-way test (Podsakoff et al., 2003) to conduct a *post-hoc* statistical test for common method bias. The results showed that a total of 19 factors had an eigenroot greater than 1, and the variation explained by the first factor was 24.99%, which was less than the critical value of 40%, indicating that the common method bias was not significant (Tang and Wen, 2020).

### 3.2 Diagnosis of multicollinearity

If the tolerance is less than 0.1 or the variance inflation factor (VIF) is more than 10, the independent variables may have multicollinearity. In this study, the tolerance values and the VIF values of each variable ranged from 0.708 to 0.779 and from1.284 to 1.413, respectively, indicating that there is no problem of multicollinearity among the variables, which will not affect the interpretation of the results of the regression analysis.

### 3.3 Descriptive statistics and correlation analysis

Table 1 shows descriptive statistics (i.e., mean and standard deviation) of variables. In addition, the results of the correlation matrix between variables are shown. The results of the study can be visualized quite well: Procrastination showed a significant negative correlation with time management disposition, motivation to exercise, and physical activity, and a significant positive correlation was found between time management disposition, motivation to exercise, and physical activity.

**Table 1:** Descriptive statistics and correlation analysis results for each variable.

		M (SD)	1	2	3	4	5	6	7	8	9	10	11	12
1	Procrastination	50.602 (9.289)	1	−0.491^**^	−0.324^**^	−0.484^**^	−0.494^**^	−0.406^**^	−0.405^**^	−0.331^**^	−0.379^**^	−0.343^**^	−0.388^**^	−0.251^**^
2	Time management disposition	147.585 (20.808)	−0.491^**^	1	0.792^**^	0.956^**^	0.926^**^	0.406^**^	0.393^**^	0.388^**^	0.361^**^	0.323^**^	0.383^**^	0.257^**^
3	The sense of time value	36.448 (5.9)	−0.324^**^	0.792^**^	1	0.607^**^	0.652^**^	0.454^**^	0.434^**^	0.384^**^	0.428^**^	0.379^**^	0.441^**^	0.161^**^
4	The sense of time control	77.462 (11.786)	−0.484^**^	0.956^**^	0.607^**^	1	0.861^**^	0.313^**^	0.305^**^	0.326^**^	0.266^**^	0.240^**^	0.289^**^	0.254^**^
5	The sense of time efficacy	33.675 (5.257)	−0.494^**^	0.926^**^	0.652^**^	0.861^**^	1	0.396^**^	0.383^**^	0.374^**^	0.355^**^	0.314^**^	0.375^**^	0.265^**^
6	Exercise motivation	59.268 (10.578)	−0.406^**^	0.406^**^	0.454^**^	0.313^**^	0.396^**^	1	0.918^**^	0.855^**^	0.945^**^	0.905^**^	0.927^**^	0.230^**^
7	Health motivation	11.989 (2.27)	−0.405^**^	0.393^**^	0.434^**^	0.305^**^	0.383^**^	0.918^**^	1	0.713^**^	0.852^**^	0.808^**^	0.810^**^	0.217^**^
8	Ability motivation	11.377 (2.307)	−0.331^**^	0.388^**^	0.384^**^	0.326^**^	0.374^**^	0.855^**^	0.713^**^	1	0.751^**^	0.690^**^	0.738^**^	0.229^**^
9	Fun motivation	11.964 (2.228)	−0.379^**^	0.361^**^	0.428^**^	0.266^**^	0.355^**^	0.945^**^	0.852^**^	0.751^**^	1	0.826^**^	0.877^**^	0.196^**^
10	Appearance motivation	11.827 (2.399)	−0.343^**^	0.323^**^	0.379^**^	0.240^**^	0.314^**^	0.905^**^	0.808^**^	0.690^**^	0.826^**^	1	0.787^**^	0.203^**^
11	Social motivation	12.111 (2.421)	−0.388^**^	0.383^**^	0.441^**^	0.289^**^	0.375^**^	0.927^**^	0.810^**^	0.738^**^	0.877^**^	0.787^**^	1	0.203^**^
12	Physical activity	15.911 (16.063)	−0.251^**^	0.257^**^	0.161^**^	0.254^**^	0.265^**^	0.230^**^	0.217^**^	0.229^**^	0.196^**^	0.203^**^	0.203^**^	1

^**^p < 0.01.

### 3.4 The relationship between procrastination and physical activity among college students: a chain mediation effect test

To test the hypothesized model, the SPSS PROCESS macro developed by Hayes was used to conduct a chained mediation effect analysis, and model 6 was selected for the chained mediation effect test (Hayes, 2018). Bootstrap sampling was set to repeat the extraction 5,000 times to test the significance of the mediation effect, based on whether the 95% confidence interval contained 0. If the confidence interval did not contain 0, it meant that the mediation effect was significant, and if it contained 0, it meant that the mediation effect was not significant, and the mediation effect of time management disposition and exercise motivation between procrastination and physical activity level was analyzed using gender and age as control variables.

**Figure 2:**
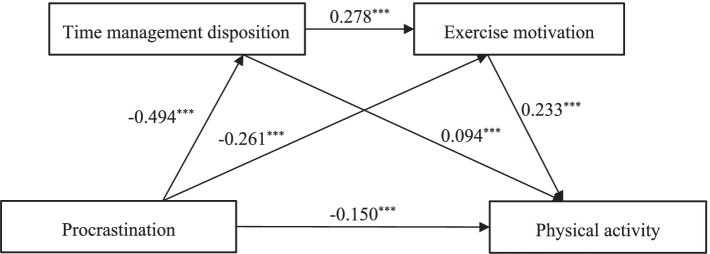
Model diagram of the effect of time management disposition and exercise motivation. ^***^p < 0.01.

As shown in Table 2 and Figure 2, procrastination significantly negatively predicted physical activity (*β* = −0.271, *p* < 0.001); procrastination significantly negatively predicted time management disposition (*β* = −0.494, *p* < 0.001), and time management disposition significantly and positively predicted physical activity (*β* = 0.094, *p* < 0.001); procrastination significantly and negatively predicted exercise motivation (*β* = −0.261, *p* < 0.001), and time management disposition significantly positively predicted exercise motivation (*β* = 0.278, *p* < 0.001); when procrastination, time management disposition, and exercise motivation were entered into the equation at the same time, procrastination significantly negatively predicted physical activity (*β* = −0.150, *p* < 0.001), and both time management disposition and exercise motivation positively predicted physical activity (*β* = 0.094, *p* < 0.001; *β* = 0.233, *p* < 0.001).

**Table 2:** Regression analysis of the chain-mediated model.

Regression equation variable		Overall fit index of the equation			Significance of regression coefficients		
Outcome variable	Predictor variable	*R*	*R* ^2^	*F*	*β*	SE	*t*
Physical activity		0.355	0.126	68.862^***^			
	Gender				−0.252	0.985	−8.313^***^
	Procrastination				−0.271	0.053	−8,930^***^
Time management disposition		0.492	0.242	152.460^***^			
	Gender				−0.038	1.188	−1.332
	Procrastination				−0.494	0.063	−17.462^***^
Exercise motivation		0.482	0.232	95.974^***^			
	Gender				0.106	0.609	3.718^***^
	Procrastination				−0.261	0.037	−7.965^***^
	Time management disposition				0.278	0.017	8.520^***^
Physical activity		0.406	0.165	46.894^***^			
	Gender				−8.513	0.972	−8.760^***^
	Procrastination				−0.150	0.061	−4.260^***^
	Time management disposition				0.094	0.027	3.442^***^
	Exercise motivation				0.233	0.051	4.534^***^

The values of each variable in the model were standardized before being substituted into the equation; ^***^p < 0.001.

The results show (Table 3) that after controlling for gender, procrastination was able to exert an effect on physical activity not only through the separate mediating effects of time management disposition and exercise motivation but also through the chained mediating effect of time management disposition → exercise motivation (none of the three paths of the mediating effect had Bootstrap 95% confidence intervals that included 0). Specifically, the mediating effect works in the effect of procrastination on physical activity through three paths: (1) indirect path 1 (procrastination → time management disposition → physical activity), with an effect value of −0.060, accounting for 22.14% of the total effect; (2) indirect path 2 (procrastination → exercise motivation → physical activity), with an effect value of −0.040, accounting for 14.76% of the total effect; (3) indirect path 3 (procrastination → time management disposition → exercise motivation → physical activity), with an effect value of −0.039, accounting for 7.75% of the total effect. The total mediated effect value was −0.021, accounting for 44.65% of the total effect.

**Table 3:** Chained mediation model effect test.

Effect	Pathway relationship	Efficiency value	95% confidence interval	Percentage of total effect
Total effect		−0.271^***^	[−0.331, −0.212]	
Total indirect effect		−0.121^***^	[−0.162, −0.081]	44.65%
Direct effect	Procrastination → physical activity	−0.260^***^	[−0.379, −0.140]	
Path 1	Procrastination → time management disposition →physical activity	−0.060^***^	[−0.103, −0.017]	22.14%
Path 2	Procrastination → exercise motivation → physical activity	−0.040^***^	[−0.061, −0.021]	14.76%
Path 3	Procrastination → time management disposition → exercise motivation → physical activity	−0.021^***^	[−0.033, −0.011]	7.75%
Comparison 1	Path 1 – Path 2	−0.020	[−0.075, 0.034]	
Comparison 2	Path 1 – Path 3	−0.039	[−0.086, 0.009]	
Comparison 3	Path 2 – Path 3	−0.019	[−0.038, −0.003]	

^***^p < 0.001.

The results of the two-by-two comparison of the indirect effects of the different mediating pathways are shown (Table 3): No significant differences were found between the separate mediating effect of time management disposition and the separate mediating effect of exercise motivation and the chained mediating effect of time management disposition → exercise motivation (Bootstrap 95% confidence intervals for Comparison 1 and Comparison 2 included 0). The separate mediating effect of exercise motivation was significantly higher than the chained mediating effect of time management disposition → exercise motivation (Bootstrap 95% confidence intervals for Comparison 3 did not include a value of 0).

## 4 Discussion

Based on the self-determination theory, the procrastination-health model, and the mechanism model of exercise persistence, this study investigated the effects of procrastination on college students’ physical activity and revealed the mechanisms by which procrastination affects physical activity through intrinsic psychological factors (time management disposition and exercise motivation). The results of the study further reveal the internal mechanisms by which procrastination affects physical activity among college students and can provide suggestions and guidance for improving procrastination and promoting physical activity among college students.

### 4.1 Characteristics of procrastination, time management disposition, exercise motivation, and current status of physical activity among college students

The study examined the effects of procrastination, time management disposition, and exercise motivation on physical activity among Chinese college students based on a health-related model combined with a maturation scale. The results of the descriptive statistics show that Chinese college students currently have a high overall level of procrastination but a high degree of time management, and although they have strong exercise motivation, the amount of physical activity is low, which is a worrying situation. It can be seen that college students are able to manage and utilize their time more reasonably and have a tendency to participate in sports activities, but there are certain problems in the process of transforming exercise motivation into actual physical activity behavior accompanied by a certain procrastination tendency. This is more consistent with existing research (Wang, 2016). In addition, even if college students want to participate in sports activities for some reason, the diversified recreational facilities brought about by the rapid development and popularization of information technology and intelligent technology, as well as the global pandemic of the new crown epidemic in recent years, are also important factors that lead to the limitation of the transformation of exercise motivation into actual sports activities.

### 4.2 The role of college student procrastination in cutting down on physical activity

The results of the study show that there is a significant negative correlation between college students’ procrastination and their participation in sports activities, that is, the higher the degree of procrastination, the fewer college students participate in sports activities, and the results of the regression analysis further proved that procrastination has a significant negative predictive effect on exercise behavior, which is consistent with the research hypothesis. From a practical point of view, procrastination, as a kind of delayed behavioral tendency, acts as an obstacle factor in the transition from “knowing” to “doing.” The deeper the degree of procrastination, the deeper the degree of procrastination, leading to a decline in individual physical activities, such as physical exercise, leisure activities, social interaction, and other behaviors. The deeper the obstacles, the more the individual’s physical activities such as physical exercise, leisure activities, and social interactions. According to the theory of planned behavior, individual behavior is influenced by behavioral intentions, attitudes, subjective norms, and a sense of behavioral control (Ajzen, 2011). In the context of college students, engaging in physical activity behavior requires a combination of factors, such as the individual’s cognitive attitude toward exercise, their grasp of the difficulty of carrying out the exercise, and external evaluative pressures regarding their own exercise activities. Research has shown that procrastination negatively correlates with an individual’s willingness to engage in physical activity (Sirois, 2004). In other words, although college students may have positive attitudes toward physical activity and be encouraged by external environmental factors, as well as have the ability to carry out the behavior, they may still be affected by procrastination, which may diminish their willingness to exercise. From the perspective of time discounting, individuals with procrastination tendencies are more likely to succumb to short-term temptations than to commit themselves to volitionally demanding tasks, so procrastinators are more likely to choose long-term volitional goals such as recreation and leisure that provide instant gratification, as well as physical activities (Song et al., 2015). Therefore, if there are more relaxing ways of leisure in daily life, college students may procrastinate or just give up participating in sports and join other recreational activities, which may be an important reason why procrastination directly affects college students’ sports activities.

### 4.3 Mediating effects of time management disposition and exercise motivation

It has been found that procrastination can influence physical activity through the separate mediating effects of time management disposition and exercise motivation and the chain mediating effect of time management disposition-exercise motivation, respectively. Time management involves a series of episodic behaviors such as setting goals, making plans, assigning priorities, allocating time, and providing feedback on results and is a psychological and behavioral characteristic of individuals in their approach to the function and value of time and the way they use it (Macan et al., 1990). Individuals with higher time management disposition tend to be able to allocate their time and tasks appropriately, which, in turn, leads to positive physical activity; conversely, individuals with lower time management disposition will increase the development of maladaptive behaviors and psychological problems, such as problematic cell phone use and academic anxiety (Zhang et al., 2021). At the same time, some researchers believe that procrastination is an externalized manifestation of a failure of self-regulation and a decline in self-control (Li et al., 2020; Deci and Ryan, 2008) and that individuals with procrastination have perceptions that time is of little value, that time does not need to be monitored, and that this negatively affects time management disposition. This shows that college students with higher levels of procrastination possess lower levels of time management disposition and therefore lower physical activity participation, that is, time management disposition mediates between procrastination and physical activity (Hypothesis H2 holds). In addition to this, it was found that procrastination can have an effect on physical activity through the mediating role of exercise motivation (Hypothesis H3 holds). According to self-determination theory, individuals are innately endowed with the expectation of self-actualization and development, and their behavioral motivation is a dynamic continuum from outside to inside (Deci and Ryan, 2008). Previous research has similarly identified the important mediating role of exercise motivation in the influence of many individual traits, such as self-enhancement (Zhou et al., 2022), exercise identity (Dong and Mao, 2020a), and perfectionism (Dong and Mao, 2020b), on physical activity, and has also been recognized as an important determinant of exercise motivation. Therefore, exercise motivation, as an important psychological resource for promoting physical activity, can effectively alleviate the lack of physical activity participation caused by negative individual traits such as procrastination. It can be seen that ameliorating procrastination tendencies is an effective strategy to cultivate good exercise habits among college students, which can effectively stimulate the degree of self-determination of exercise motivation and promote physical activity among college students to a certain extent.

This study also found that procrastination can influence college students’ physical activity through the chain mediation of time management disposition → exercise motivation (Hypothesis H4 holds). It has been found that individuals with stronger time management disposition and motivation to exercise tend to have higher levels of physical activity (Li et al., 2021), both of which are psychologically motivating factors for individuals to engage in physical activity and exercise adherence (Guo and Mao, 2023), and can be influenced by individual characteristics. Higher procrastination leads individuals to exhibit higher levels of discretion and inertia in their behavior in response to events, resulting in less self-determined and more non-self-determined levels of motivation for physical activity, and may even persist in an unmotivated state of participation. At the same time, there is a positive relationship between time management disposition and individual motivation; individuals with a stronger time management disposition tend to have higher achievement motivation. Procrastination can negatively impact exercise motivation by decreasing time management disposition, which is not conducive to college students’ participation in physical activity (Wang et al., 2023). Thus, procrastination is also able to influence exercise behavior through the chain-mediated effects of time management disposition and exercise motivation.

### 4.4 Practical significance

This study explored in depth the relationship between college students’ procrastination and physical activity, examined the internal role mechanism of time management disposition and exercise motivation in the impact of procrastination on physical activity, constructed a chain mediation model of college students’ physical activity influencing factors, and provided a practical basis for reducing the occurrence of college students’ procrastination and promoting active participation in physical activity. According to the results of the study, educational authorities and educators should strengthen the cultivation of college students’ time management ability and utilize the aggregation effect of society, family, school, peers, and other multi-party systems to build a good ecology of sports activities and to stimulate college students’ motivation to participate in sports activities. As college students at an important stage of their lives, they should take the initiative to overcome and improve procrastination and other undesirable behaviors, improve their ability to plan their time rationally, actively participate in and adhere to sports activities by adopting goal-setting and other methods, and establish a sense of “lifelong sports,” to promote the all-around healthy development of the body and mind.

### 4.5 Research shortcomings and prospects

There are some shortcomings in this study. First, the use of a self-reporting scale to assess procrastination and time management disposition may have the situation that there is a discrepancy between self-evaluation and the actual behavior of the subjects due to their strict demands on the self. Second, in response to the COVID-19 pandemic, governments generally adopted closure measures, and although this had no significant impact on the collection of our questionnaires, this unexpected event objectively reduced the amount of time people spent participating in physical activity, which may have had an impact on the results of this study. Future studies might be able to conduct a longitudinal study using pre-pandemic control group data to reveal the specific impact of this event on the results of the study. In addition, in terms of measurement, more objective measures such as accelerometers could be used in the future to compensate for the lack of self-reported data.

## 5 Conclusion

In conclusion, this study has shown that procrastination can significantly and negatively predict physical activity among Chinese university students. Time management disposition and exercise motivation can individually mediate the effect of procrastination on physical activity. Procrastination can have an effect on Chinese university students’ physical activity through the chain mediation of time management disposition and exercise motivation.

Based on the research findings discussed above, this study offers the following recommendations for improving physical activity among university students: First, it is important to address the adverse effects of procrastination among college students. Regular health education can be conducted by means of relevant courses to enhance their understanding of the harms associated with procrastination and to cultivate their ability to manage and apply time scientifically. Second, it is essential to enhance the construction of places for physical activities, promote healthy lifestyles and sports concepts, provide convenient exercise resources and guidance for college students, increase their motivation to exercise, and promote their active participation in physical activities.

## Data Availability

The datasets presented in this article are not readily available because the data presented in this study are available on request from the corresponding author. These data are not publicly available because they are part of an ongoing study, which requires maintaining confidentiality and integrity to ensure the validity of future, more in-depth research findings. Requests to access the datasets should be directed to yangjianxz@sina.com.
